# Advection surface-flux balance controls the seasonal steric sea level amplitude

**DOI:** 10.1038/s41598-024-61447-y

**Published:** 2024-05-09

**Authors:** Antoine Hochet, William Llovel, Thierry Huck, Florian Sévellec

**Affiliations:** 1 Univ Brest, CNRS, IFREMER, IRD, Laboratoire d’Océanographie Physique et Spatiale (LOPS, UMR 6523), IUEM, Brest, France; 2 INRIA, CNRS, ODYSSEY Team-Project, Brest, France

**Keywords:** Seasonal steric sea level, Physical mechanisms, Surface heat fluxes, Ocean advective processes, Ocean sciences, Physical oceanography, Climate sciences, Ocean sciences, Physical oceanography

## Abstract

Along with the mean sea level rise due to climate change, the sea level exhibits natural variations at a large number of different time scales. One of the most important is the one linked with the seasonal cycle. In the Northern Hemisphere winter, the sea level is as much as 20 cm below its summer values in some locations. It is customary to associate these variations with the seasonal cycle of the sea surface net heat flux which drives an upper-ocean thermal expansion creating a positive steric sea level anomaly. Here, using a novel framework based on steric sea level variance budget applied to observations and to the Estimating the Circulation and Climate of the Ocean state estimate, we demonstrate that the steric sea level seasonal cycle amplitude results from a balance between the seasonal sea surface net heat flux and the oceanic advective processes. Moreover, for up to 50% of the ocean surface, surface heat fluxes act to damp the seasonal steric sea level cycle amplitude, which is instead forced by oceanic advection processes. We also show that eddies play an important role in damping the steric sea level seasonal cycle. Our study contributes to a better understanding of the steric sea level mechanisms which is crucial to ensure accurate and reliable climate projections.

## Introduction

Global mean sea level (GMSL) rise is one of the most emblematic and objective consequences of current global warming. The rate of GMSL rise reached 3 mm per year over the 1993–2015 period^1^. However, this trend is modulated by a seasonal signal which reaches a magnitude of 4 mm at the end of boreal summer^2^, based on satellite altimetry data. The main drivers of GMSL rise are global ocean mass increase (i.e., barystatic sea level^3^) and global ocean density change (global mean steric sea level). The latter is primarily controlled by global ocean warming. Although global mean sea level is highly relevant as a climate index, coastal population are more affected by regional sea level variations. These regional variations are usually much larger than the yearly increase of GMSL. Locally, the sea level seasonal cycle can indeed have amplitudes up to ${20}\,\hbox {cm}$ in some regions^4^, and are thus comparable to the magnitude of the GMSL rise over the last century^5,6^. It is therefore of primary importance to understand their mechanisms, in order to ensure that they are correctly represented in climate models and that future estimates are accurate.

**Figure 1:**
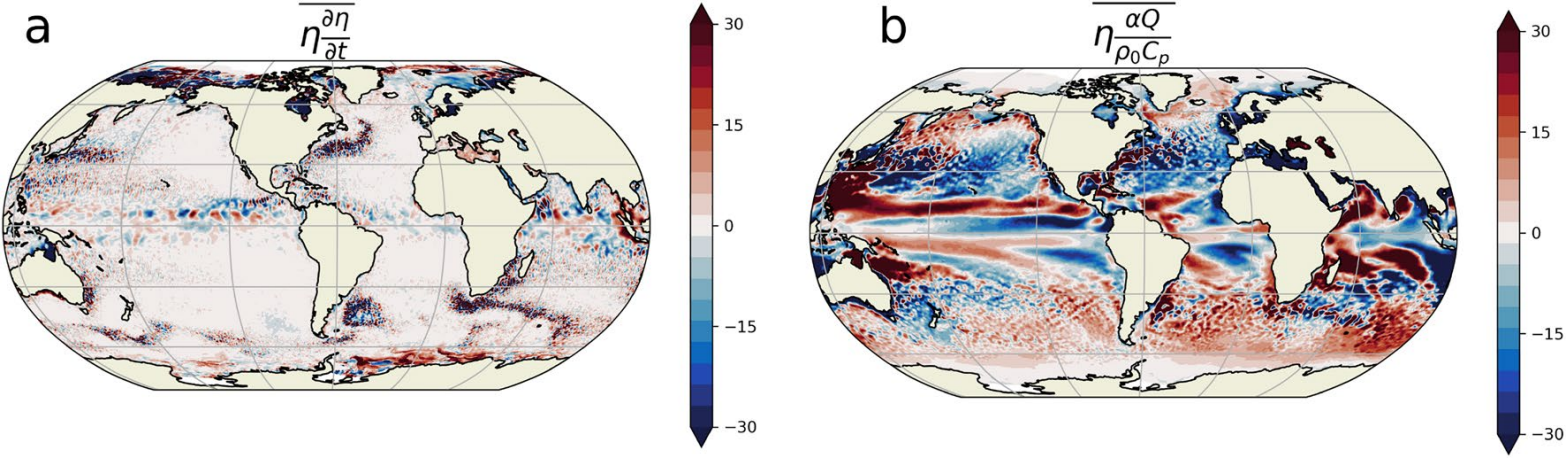
Observations reveal that the sea level seasonal cycle cannot be solely explained by surface net heat flux variations. Time average (1993–2014) of the product between the two terms of Eq. (1) and seasonal variations in SSL ($\eta$): (**a**) left hand side (i.e. $\overline{\eta \frac{\partial \eta }{\partial t}}$) (in $\hbox {cm}^{2}\,\hbox {year}^{-1}$) where $\eta$ is approximated by satellite altimetry^7^ and (**b**) right hand side (i.e. $\overline{\eta \frac{\alpha Q}{\rho _0 C_p}}$) (in $\,\hbox {cm}^{2}\,\hbox {year}^{-1}$) where *Q* is obtained from the ERA5 reanalysis. The lhs term (**a**) is different from the rhs term (**b**), thus demonstrating that important terms are missing in Eq. (1).

**Figure 2:**
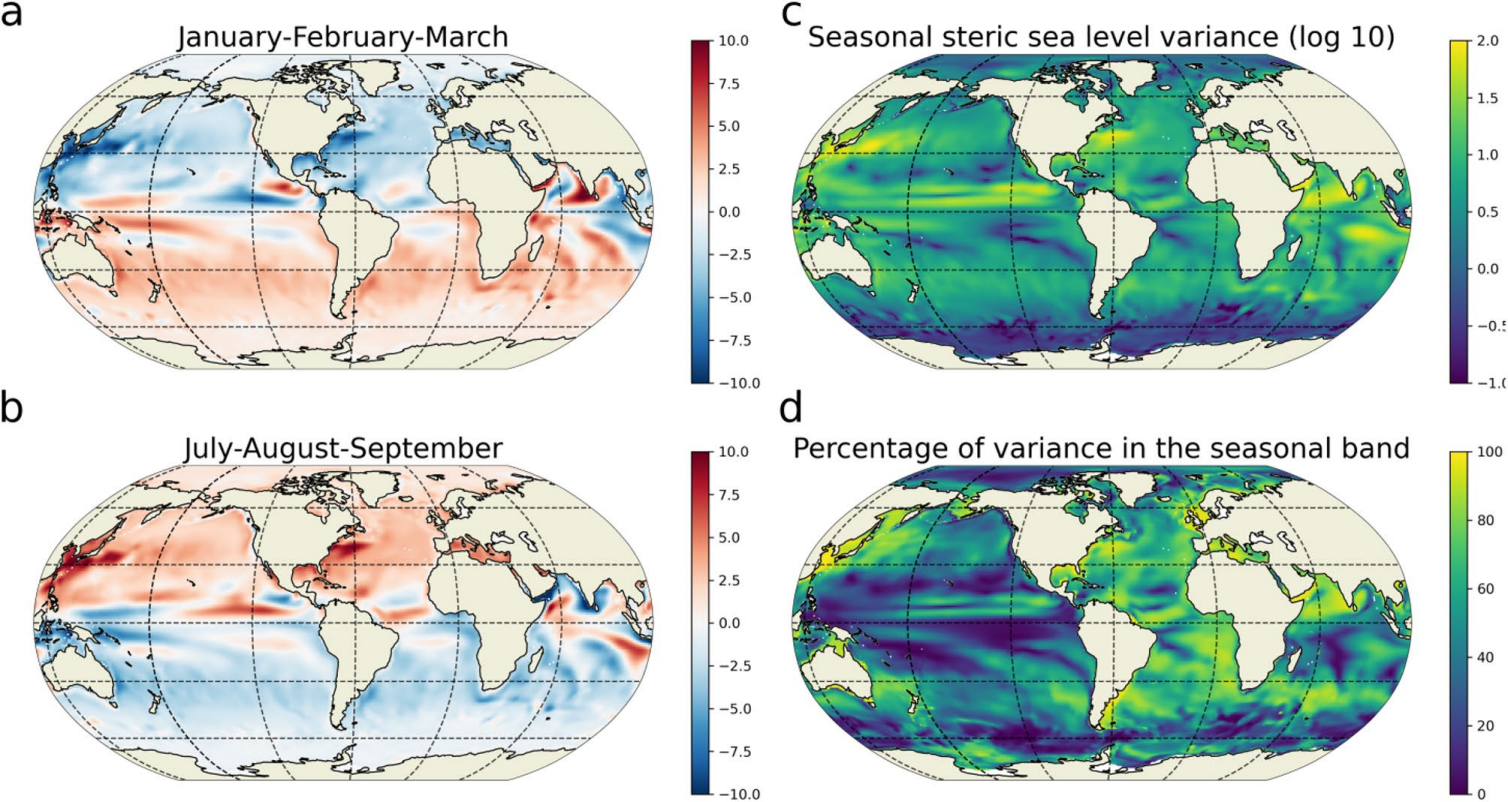
Seasonal SSL cycle in ECCOv4r3 1993–2014. (left) Seasonal SSL anomaly (in cm) averaged for January–February–March (**a**) and July–August–September (**b**). (right) Log10 of the variance ($\hbox {cm}^{2}$) of the seasonal SSL anomalies in ECCOv4r3 over the 1993–2014 period (**c**) and percentage of the total variance explained by the seasonal cycle (**d**).

Once corrected for the atmospheric pressure effect, these regional sea level variations are mostly associated with steric sea level (SSL) variations: they owe their existence to the seasonal variations of the oceanic density field^8^. Previous studies based on the scale analysis of the SSL variations equation^8–12^ have found that these variations are mainly driven by the seasonal variations of net heat flux to the ocean. During the Northern and Southern Hemisphere summer months, this anomalous net heat flux (i.e., “warming”) leads to a thermal expansion of the water column inducing a positive anomaly of SSL. Changes in the steric component of the sea level $\eta$ are assumed to be given at first order by^10^:

1 $$\begin{aligned} \partial _t \eta \approx \frac{\alpha Q}{\rho _0 C_p} \, , \end{aligned}$$

where *Q* is the anomalous sea surface net heat flux, $\alpha$ the thermal expansion coefficient, $\rho _0$ the reference density, and $C_p$ the specific heat of seawater. The reconstruction of the SSL from the time integration of Eq. (1) is generally found to be well correlated with the true SSL everywhere except at low latitudes^10^, where the effect of wind and baroclinic Rossby wave propagation needs to be included^13–19^. These positive correlations are often interpreted as the signature of the leading role of the sea surface seasonal heat flux anomalies in driving the seasonal SSL variations^4,8,10,12,20–24^. The underlying assumption behind Eq. (1) is that the SSL seasonal cycle is a passive local response of the ocean to the seasonal cycle in surface net heat flux. Multiplying the SSL time variations equation by $\eta$ yields an equation for the time tendencies of $\eta$ squared i.e. for the amplitude of the seasonal cycle. This equation allows us to determine which of the terms are acting to increase or decrease the amplitude of the cycle. Considering Eq. (1), if $\overline{\eta \frac{\alpha Q}{\rho _0 C_p}}>0$, the amplitude increases and if $\overline{\eta \frac{\alpha Q}{\rho _0 C_p}}<0$, the amplitude decreases (where the over bar represents the time mean). As Eq. (1) has only two terms, they should remain equal when multiplied by $\eta$. However, we show in Fig. 1, using a combination of satellite altimetry (AVISO^7^) and surface net heat flux from the ECMWF ERA5 reanalysis^25^ that, when multiplied by $\eta$ and time averaged over the period 1993–2014, the left hand side term of Eq. (1) is not equal to its right hand side term as it should be if Eq. (1) was complete (see “Methods” for more details about this calculation). Moreover, the magnitude of the right hand side term is around ${30}\,\textrm{cm}^{2}\,\textrm{year}^{-1}$ (Fig. 1b) everywhere while the SSL variance magnitude is around ${100}\,\textrm{cm}^{2}$ (see Fig. 2c). It follows that without any other term in Eq. (1) a time scale of 3 to 4 years ($100\,\hbox {cm}^{2}\big /30\,\hbox {cm}^{2}\,\hbox {year}^{-1}$) would be sufficient to completely modify the present SSL seasonal cycle. Additionally, Fig. 1b shows that large regions of the ocean have negative values of $\overline{\eta \frac{\alpha Q}{\rho _0 C_p}}$ suggesting that the amplitude of the SSL seasonal cycle is, in large parts of the ocean and across all latitudes, damped by the seasonal heat flux, implying that several important terms are missing in Eq. (1). This strongly contrasts with the Sea Surface Temperature (SST) seasonal cycle amplitude which is virtually everywhere forced by the seasonal net heat flux i.e. $\overline{\frac{SST Q}{\rho _0 C_p}}>0$ (see Fig. S1 of the supporting information) as already noticed by several authors^17,26^. Understanding what are the missing term(s) in the equation controling the amplitude of the SSL seasonal cycle and explaining why the atmospheric net heat flux can dampen the SSL seasonal cycle in some regions are the two main objectives of this work.

While the interannual variability and long-term trend of SSL have been often investigated in the previous years^27–30^, the seasonal cycle of SSL has received much less attention. Hence, here, we apply for the first time a powerful diagnostic to characterize the drivers of the SSL seasonal cycle and describe the dynamics of its sources and sinks. This diagnostic is based on steric sea level variance budget. It has recently been developed and applied to interannual steric sea level variations to understand their mechanisms^30^. Variance budgets are a common tool in physics and have been widely applied to various variables (density, temperature, salinity) in the oceanographic literature in the past^31–37^. This diagnostic is constructed in a similar way to the kinetic energy budget, and is a rigorous way to assess the mechanisms controlling the steric sea level variability.

## Budget of seasonal SSL variance

To investigate the balance controlling the amplitude of the seasonal cycle of regional SSL, we compute the budget of variance over 1993–2014 based on the ECCOv4r3 state estimate^38^. The seasonal cycle of each variable is obtained from the time mean of the 22 years monthly time series (see “Methods”). At mid and high latitudes, in the Northern (Southern) Hemisphere, the seasonal SSL anomaly is negative (positive) ($\sim {10}\,\hbox {cm}$) in boreal winter while it is positive (negative) in summer (Fig. 2a,b). Close to the equator in each hemisphere, the anomaly can be positive or negative over the two periods of time, depending on the location. Western boundary currents, such as the Kuroshio and the Gulf Stream, also have a strong signature in SSL seasonal cycle. The largest values of the seasonal SSL variance are of the order of $10^{2}\,\hbox {cm}^{2}$ (Fig. 2c) and are found at low latitude in the Eastern Pacific, in the North Indian Ocean and the South Equatorial Current regime of the Indian Ocean and in the northern hemisphere western boundary currents. The seasonal SSL variance represents a large proportion (i.e., above 50%) of the total variance in numerous regions (Fig. 2d). This is particularly true in the Atlantic Ocean across all latitudes and in western boundary currents of the Pacific Ocean as well as in the Arabian Sea and western Bay of Bengal.

**Figure 3:**
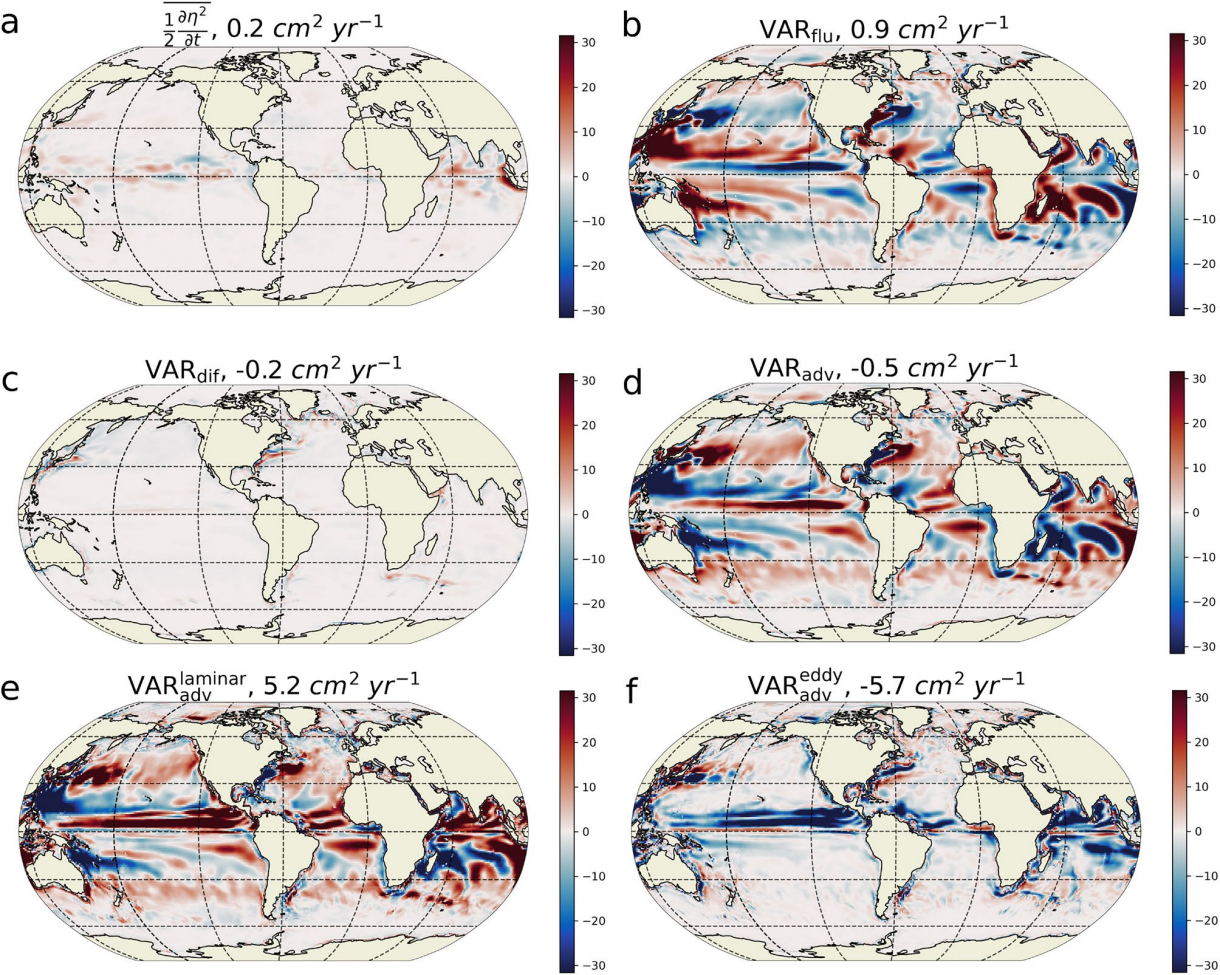
Seasonal SSL variance budget. (**a**) Time mean of the time tendencies of the SSL seasonal variance (in $\hbox {cm}^{2}\,\hbox {year}^{-1}$). (**b**) Contribution of the ocean surface buoyancy fluxes. (**c**) Contribution of the diffusive terms on the budget. (**d**) Contribution of the advective term. Positive values (red) indicates that the corresponding term acts to increase the seasonal SSL variance, negative values (blue) that it acts to decrease the variance. The advective term ($\mathrm {VAR_{adv}}$) is further decomposed into the advection by (**e**) the resolved “laminar” velocities ($\mathrm {VAR^{laminar}_{adv}}$) and (**f**) the eddy-induced velocities from the GM parameterization ($\mathrm {VAR^{eddy}_{adv}}$). The global average of each term is given in the corresponding titles.

The variance budget for the seasonal cycle of the SSL ($\eta$) is composed of four different terms (see section “SSL variance budget” in “Methods”):

2 $$\begin{aligned} \overline{ \frac{1}{2}\frac{\partial \eta ^2}{\partial t}}= & {} \mathrm {VAR_{adv}} +\mathrm {VAR_{dif}} +\mathrm {VAR_{flu}}. \end{aligned}$$

The overline represents the time average over the ECCOv4r3 period, the left hand side term is the averaged time evolution variance of the seasonal cycle of the SSL . On the right hand side, $\mathrm {VAR_{adv}}$ is the effect of the oceanic advective terms, $\mathrm {VAR_{dif}}$ the effect of parameterized diffusion (including isoneutral and dianeutral mixing), convective adjustment and background vertical mixing, and $\mathrm {VAR_{flu}}$ is the effect of the seasonal cycle of the ocean surface buoyancy fluxes. For instance, a positive $\mathrm {VAR_{flu}}$ indicates a local source of SSL variance because it acts to increase the SSL variance (i.e. the SSL amplitude). On the contrary, if it is negative, it acts to decrease the variance: it is a sink of SSL variance. The most striking feature in the budget (Fig. 3) is the strong local compensation between the two dominant terms, the buoyancy forcing term ($\mathrm {VAR_{flu}}$) and the advective term ($\mathrm {VAR_{adv}}$), both reaching values up to $\pm {30}\,\hbox {cm}^{2}\,\hbox {year}^{-1}$. It is shown in the supplementary information file that $\mathrm {VAR_{flu}}$ is largely dominated by the net heat flux, in agreement with the literature (Fig. S2). On the other hand, the diffusive term ($\mathrm {VAR_{dif}}$) is approximately one order of magnitude smaller (bounded by $\pm \,\hbox {cm}^{2}\,\hbox {year}^{-1}$), and can then be ignored in the overall main balance of the cycle. The term associated with the time tendency of the seasonal cycle SSL variance is also an order of magnitude smaller than the other terms, but is not exactly zero. This is explained by the methodology used to extract the seasonal cycle as explained in section “Methods”. This term remains much smaller than the dominant terms of the budget and can be neglected. Figure 3 demonstrates that the main balance in the seasonal SSL variance budget is between the advective and buoyancy forcing terms. It contrasts strongly with Eq. (1), from which a constant amplitude could only be attained if $\overline{\eta Q}=0$, which is clearly not the case in Fig. 1b as well as in Fig. 3b. The fact that $\overline{\eta Q}\ne 0$ implies that $\frac{\partial \eta }{\partial t}$ is not exactly in phase with *Q*. The time lag between these two terms is estimated to be between 2 and 3 weeks depending on the location (supplementary file Fig. S3). Although this time lag may seem small, it represents between 15% and 25% of the time lag required for these two terms to be in quadrature, which is about 13 weeks (i.e. a quarter of an annual period). This relatively small time lag is sufficient to induce the large values of $\mathrm {VAR_{flu}}$ found in Figs. 1b and 3b. Moreover, the seasonal cycle of the ocean surface buoyancy fluxes acts as a sink of seasonal SSL variance over 49% of the ocean surface. In these regions, the seasonal cycle of the buoyancy flux damps the seasonal cycle of SSL instead of sustaining it in regions where it is positive. The locations where $\mathrm {VAR_{flu}}$ acts as a sink are relatively symmetric with respect to the equator: they are found between ${30}^\circ \,\hbox {N}$ and ${60}^\circ \,\hbox {N}$ and between ${30}^\circ \,\hbox {S}$ and ${60}^\circ \,\hbox {S}$ in every oceans, in the eastern half of the Pacific, Atlantic, and Indian Oceans at low latitudes. Very similar patterns are found using ERA5 and satellite altimetry (Fig. 1b) in the eddy-rich regions, such as the Southern Ocean, which are not resolved by the laminar resolution of the ECCO v4. We find that the variance budget is very similar when all frequencies resolved by ECCO v4 are considered (i.e. when the mean seasonal cycle is not extracted) (see Fig. S4 of the supporting information). This first shows that the SSL variance fluxes linked with the mean seasonal cycle dominate the SSL variance budget at all frequencies and secondly demonstrates that our results do not depend on the methodology used to extract the mean seasonal cycle. The horizontal average of each term, given in the titles of Fig. 3, shows that globally, the surface buoyancy flux is a source (${0.9}\,\hbox {cm}^{2}\,\hbox {year}^{-1}$) that is partially balanced by advection (${-0.5}\,\hbox {cm}^{2}\,\hbox {year}^{-1}$) over the period 1993–2014 (global mean diffusion is ${-0.2}\,\hbox {cm}^{2}\,\hbox {year}^{-1}$, being a net sink, and the variance time tendency is ${0.2}\,\hbox {cm}^{2}\,\hbox {year}^{-1}$).

**Figure 4:**
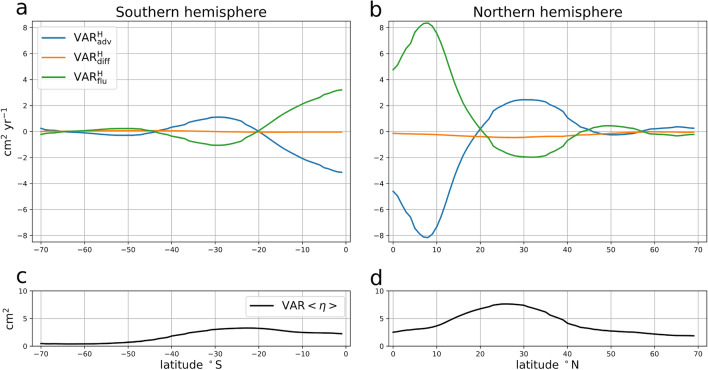
Variance budget for the spatially averaged Seasonal SSL. (**a**) Terms of the variance budget (in $\,\hbox {cm}^{2}\,\hbox {year}^{-1}$) for the spatially averaged SSL over Southern Hemisphere regions with latitudes south of the latitude given by the abscissa value. (**b**) same but for Northern Hemisphere regions with latitudes north of the abscissa value. The Contributions of the advective term (blue line), time mean of the time tendencies of the SSL seasonal variance (orange line), diffusive terms (green line) and of the ocean surface buoyancy fluxes (red line) are shown. (**c**) Variance of the regionally averaged SSL seasonal cycle for latitudes south of the abscissa value (in $\hbox {cm}^{2}$), (**d**) Same but for the northern hemisphere (i.e. latitudes north of the abscissa value)..

To investigate the large-scale mechanisms of seasonal SSL variations, we also compute the variance equation for the hemispheric average of the SSL as follows (see “Methods”):

3 $$\begin{aligned} \frac{1}{2}\overline{ \frac{\partial \langle \eta \rangle ^2}{\partial t} }= \mathrm {VAR^H_{adv}} +\mathrm {VAR^H_{dif}} +\mathrm {VAR^H_{flu}}, \end{aligned}$$

where $\langle \eta \rangle$ is the hemispheric average of the seasonal SSL, $\mathrm {VAR^H_{adv}}$, $\mathrm {VAR^H_{dif}}$, $\mathrm {VAR^H_{flu}}$ respectively the contribution of oceanic advection, diffusion and buoyancy flux. The hemispheric averages are computed over several regions bounded by different latitudes. For the northern hemisphere the averaging region is defined by all locations with latitudes north of a given latitude, which varies from ${0}^\circ \,\hbox {N}$ to ${70}^\circ \,\hbox {N}$. Similarly, for the southern hemisphere, the averaging region is defined by all locations with latitudes south of a given latitude, which varies from ${70}^\circ \,\hbox {S}$ to ${0}^\circ \,\hbox {S}$. The results show that the large-scale seasonal SSL variations obey a similar balance as for the local variance budget, with the oceanic advective terms and the buoyancy flux term being the two main contributors to the budget in both hemispheres (Fig. 4a,b). The balance in both hemispheres is nearly symmetric around ${8}^\circ \,\hbox {N}$, although the amplitudes of the averaged SSL variance budget terms are about twice as large in the Northern Hemisphere (NH) as in the Southern Hemisphere (SH). When the region encompasses the entire hemisphere (corresponding to latitude ${0}^\circ \,\hbox {N}$ for the northern hemisphere and ${0}^\circ \,\hbox {S}$ for the southern hemisphere in Fig. 4), the main source is attributed to the buoyancy flux term, which is mainly due to net heat flux (${3}\,\hbox {cm}^{2}\,\hbox {year}^{-1}$ in the SH, ${5}\,\hbox {cm}^{2}\,\hbox {year}^{-1}$ in the NH), while the main sink is associated with the advective term (${-3}\,\hbox {cm}^{2}\,\hbox {year}^{-1}$ in the SH, ${-5}\,\hbox {cm}^{2}\,\hbox {year}^{-1}$ in the NH). Although the amplitude of the seasonal variations of the hemispheric averages of the advective term ($\langle {\textrm{adv}}\rangle$, see Eq. (14) in “Methods”), is almost 3 times weaker than that of the hemispheric averages of the surface fluxes ($\langle {\textrm{flu}}\rangle$), $\langle {\textrm{adv}}\rangle$ is almost in phase with $\langle \eta \rangle$ and can balance the effect of $\langle {\textrm{flu}}\rangle$, which is almost in quadrature with $\langle \eta \rangle$ (see Fig. S5 of the supplementary file). For a region bounded to the North by ${30}^\circ \,\hbox {S}$ for the Southern hemisphere and to the South by ${30}^\circ \,\hbox {N}$ for the Northern hemisphere, the advection term is the main source of SSL seasonal variance (${1}\,\hbox {cm}^{2}\,\hbox {year}^{-1}$ in the SH, ${2.5}\,\hbox {cm}^{2}\,\hbox {year}^{-1}$ in the NH) and is balanced by the surface buoyancy flux term. The variance of the regionally averaged SSL is between 3 and ${8}\,\hbox {cm}^{2}$ in the NH and between 1 and ${4}\,\hbox {cm}^{2}$ in the SH (Fig. 4c,d) As for the local variance budget, calculating the ratio of the regionally averaged SSL variance to the budget term values gives the time scale that would be sufficient to completely change the characteristics of the current regional SSL seasonal cycle. This time scale is less than one year for the entire NH and SH regions, and about 3 years with a southern/northern boundary located at ${30}^\circ \,\hbox {S}$ and ${30}^\circ \,\hbox {N}$. This demonstrates the importance of the balance between the advective terms and the surface buoyancy fluxes in determining the amplitude of the regional SSL seasonal cycle.

## The vertical structure of the density anomaly controls the sign of $\mathrm {VAR_{flu}}$

**Figure 5:**
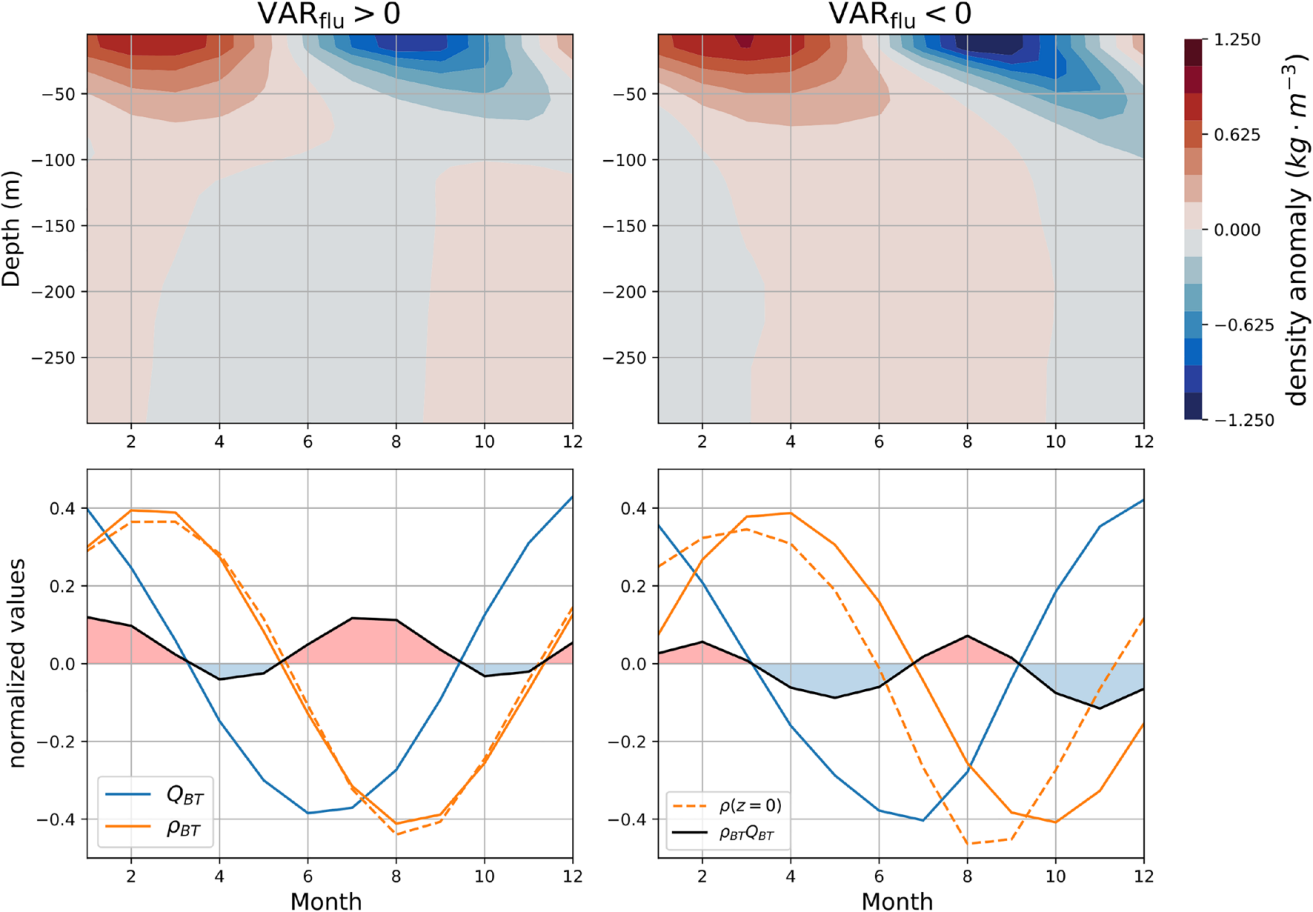
The vertical structure of density anomalies controls the sign of the surface buoyancy flux contribution to SSL variance (${\textrm{VAR}}_{\textrm{flu}}$). Top panels: density anomaly (in $\hbox {kg}\,\hbox {m}^{-3}$) as a function of depth and time (month). Bottom panels: time variation of the vertically-averaged density (orange line), vertically-averaged net heat flux (blue line) and product of the two terms, i.e., $\rho _{BT}Q_{BT}$ (black line, $\propto {\textrm{VAR}}_{\textrm{flu}}$). The surface density (orange dashed) is also shown. Each time series is normalized, red shading indicates where ${\textrm{VAR}}_{\textrm{flu}}$ is positive and blue shading where it is negative. The left and right columns show respectively the Northern Hemisphere values averaged at locations where ${\textrm{VAR}}_{\textrm{flu}}>0$ and where ${\textrm{VAR}}_{\textrm{flu}}<0$.

As shown in “Methods”, ${\textrm{VAR}}_{\textrm{flu}}$ is proportional to the product of the vertically-averaged density seasonal anomaly ($\rho _{\textrm{BT}}$) and the vertically-averaged buoyancy anomaly which is mostly due to the net heat flux ($Q_{BT}$, see Fig. S2):

4 $$\begin{aligned} \mathrm {VAR_{flu}}\propto \overline{\rho _{\textrm{BT}} Q_{BT} }, \end{aligned}$$

this formula demonstrates that the sign of ${\textrm{VAR}}_{\textrm{flu}}$ depends on the correlation between these two fields. We show (Fig. 5) the density anomaly in the first ${300}\,\hbox {m}$ as a function of time and depth, averaged North of ${20}^\circ \,\hbox {N}$ in regions where ${\textrm{VAR}}_{\textrm{flu}}>0$ (left column) and in regions where ${\textrm{VAR}}_{\textrm{flu}}<0$ (right column). We average North of ${20}^\circ \,\hbox {N}$ to ensure that the density anomalies have the same phase everywhere, but the same results hold for the Southern Hemisphere. The propagation of the density anomaly to deeper depths is not instantaneous during the seasonal cycle (top panels Fig. 5) which implies that the vertically-averaged density anomalies are not necessarily in phase with the surface density anomaly. On the contrary, the atmospheric buoyancy flux is confined to the first ${50}\,\hbox {m}$ and has a coherent phase with depth. Thus the sign of ${\textrm{VAR}}_{\textrm{flu}}$ is controlled by the vertical structure of the density anomalies. The surface density is almost everywhere positively correlated with $Q_{BT}$ (supplementary Fig. S6). However, regions where the vertically-averaged density anomaly is influenced by shifted deeper density anomalies are associated with negative values of $\mathrm {VAR_{flu}}$ (right column Fig. 5). The comparison of the time evolution of the vertically-averaged density (orange line, bottom panels Fig. 5) with the vertical integral of the seasonal buoyancy flux (blue line, Fig. 5) reveals a different behavior between regions where ${\textrm{VAR}}_{\textrm{flu}}>0$ and regions where ${\textrm{VAR}}_{\textrm{flu}}<0$. In regions where ${\textrm{VAR}}_{\textrm{flu}}$ is a source (positive), the vertically-averaged density anomaly follows the vertical integral of the seasonal cycle of buoyancy flux (blue line) with a delay of approximately two months. The time series of the product between $\rho _{BT}$ and $Q_{BT}$ is then strongly positive in boreal winter and summer (months 11 to 3 and months 6 to 9) and weakly negative for the remaining periods, resulting in an overall positive time average (${\textrm{VAR}}_{\textrm{flu}}>0$). Moreover, the vertically-averaged density is almost indistinguishable from the surface density (orange dashed line). In regions where ${\textrm{VAR}}_{\textrm{flu}}$ is a sink (negative), the vertically-averaged density anomaly is shifted away from the vertical integral of the seasonal buoyancy flux because of the influence of deeper density anomalies. The delay between the vertical integral of the seasonal cycle of buoyancy flux and the vertically-averaged density is then larger ($\sim$ 4 months) than for the previous case and induces large negative values of the product between $\rho _{BT}$ and $Q_{BT}$ in boreal spring and autumn (months 3 to 6 and months 9 to 12). Unlike the previous case, the vertically-averaged density is also shifted with respect to the surface density. It is therefore the influence of deeper density anomalies that explains why $\mathrm {VAR_{flu}}$ is a sink (negative) over large areas of the ocean.

## Eddies dampen the seasonal SSL cycle

To understand the role of eddies in ${\textrm{VAR}}_{\textrm{adv}}$ (Fig. 3d), this term is decomposed into two terms (see section “Methods”):

5 $$\begin{aligned} \mathrm {VAR_{adv}}=\mathrm {VAR^{laminar}_{adv}}+\mathrm {VAR^{eddy}_{adv}}. \end{aligned}$$

$\mathrm {VAR^{laminar}_{adv}}$ is linked to the advection of density by the resolved “laminar” currents, whereas $\mathrm {VAR^{eddy}_{adv}}$ is linked to the advection by the eddy-induced velocities as parameterized in Gent and McWilliams^39^. $\mathrm {VAR^{laminar}_{adv}}$ is important almost everywhere (Fig. 3e) and can be a source or a sink of seasonal SSL variability depending on the region. Its pattern is similar to that of $\mathrm {VAR_{adv}}$ (Fig. 3d), except around the equator and in western intensified boundary currents. When horizontally averaged, it is the largest source for the seasonal cycle (${5.2}\,\hbox {cm}^{2}\,\hbox {year}^{-1}$). At the global scale, this source is almost exactly compensated by the sink made by the term linked with eddies $\mathrm {VAR^{eddy}_{adv}}$ (${-5.7}\,\hbox {cm}^{2}\,\hbox {year}^{-1}$). The Gent & McWilliams parameterization^39^ mimics the fact that isopycnals slopes are baroclinically unstable and eddy kinetic energy is created through the release of potential energy. This eddy parameterization leads to a damping of seasonal SSL variance. Locally, $\mathrm {VAR^{eddy}_{adv}}$ (Fig. 3f) is strongly negative ($\sim {-30}\,\hbox {cm}^{2}\,\hbox {year}^{-1}$) in the equatorial regions of the Pacific, Atlantic, and Indian Oceans. There, it significantly compensates the laminar advection term. In line with our results, but for a different timescale, it has recently been shown that, in the Equatorial Pacific, ENSO variability is inhibited by mesoscale eddies^40^. It also has large values in the Gulf Stream and Kuroshio regions where it can either be a source or a sink of SSL variance.

## Discussion

Based on a new variance budget framework, we have demonstrated in this article that the amplitude of the SSL seasonal cycle is controlled both at local scale and at the hemispheric scale by a balance between the oceanic advection and surface buoyancy forcing terms. . The diffusive and variance tendency terms are an order of magnitude smaller. At mid latitudes and in the eastern parts of low latitudes regions, the seasonal cycle of the buoyancy fluxes even acts to damp the amplitude of the seasonal cycle of SSL instead of sustaining it. In these regions, the main source of SSL seasonal variability is associated with oceanic advective terms. . Whether the surface buoyancy fluxes are a source or a sink of SSL cycle variance depends on the vertical structure of the density anomalies. Buoyancy fluxes are a source of variance in regions where the vertically-averaged density is controlled by surface values of density. On the contrary, buoyancy fluxes become a sink of variance in regions where density anomalies are controlled by sub-surface density anomalies. The horizontal average of the local variance budget shows that the seasonal ocean surface buoyancy flux is a source for the seasonal cycle of SSL ($+\,\hbox {cm}^{2}\,\hbox {year}^{-1}$) , while advective terms (and diffusive terms) are a sink (respectively $-{0.5}\,\hbox {cm}^{2}\,\hbox {year}^{-1}$ and $-{0.2}\,\hbox {cm}^{2}\,\hbox {year}^{-1}$ ). We also show for the first time that eddies are a major sink of the seasonal cycle of SSL close to the equator. The horizontal resolution of the ECCOv4 reanalysis ($1^\circ$ in average) does not, however, explicitly resolve eddies and their effect is only parameterized. Further studies are thus needed to better understand their exact contribution to the seasonal cycle of SSL, in particular for western boundary currents and the Southern ocean where the turbulent field is known to imprint itself strongly on the SSL^41^. In higher resolution models, global or regional, a similar methodology could be used to study the seasonal cycle mechanisms at different space scales and determine whether different mechanisms are at play. In this work we have decomposed the oceanic advective terms into two parts: resolved and eddy induced advection. A previous study of the mechanisms of interannual variations in steric sea level has shown that the advective term can be decomposed into several terms each associated with different physical mechanisms^30^. A perspective is thus to apply the same framework to the study of the seasonal variations in SSL. Although we have shown that ECCOv4 is able to reproduce most of the patterns associated with the buoyancy flux term in the variance equation (Figs. 1a and 3b) giving us confidence in our results, a limitation of this work is the use of a single model which may be associated with substantial error model. Future work should therefore focus on reproducing our results in different numerical models. Another limitation of our study is the focus on the steric sea level. Steric sea level is the major component of sea level variations at low and mid latitudes but seasonal variations in manometric sea level are important at high latitudes and in shallow water regions. Therefore, future studies should also investigate the mechanisms of this component. One important implication of our results is that the amplitude of the sea level seasonal cycle is potentially much more sensitive to anthropogenic climate change than previously assumed. It will indeed be sensitive to changes in the advective and net heat flux seasonal cycle (whether as a source or a sink), as well as to changes in the oceanic mean circulation and eddy field. Numerous studies have demonstrated that important changes in oceanic mean circulation are underway^42^. Studying how the SSL seasonal cycle is modified by anthropogenic climate change is thus a subject of primary importance and we hope that the methodology developed in this work will help to shed light on this matter.

## Methods

### Assessment of the SSL variance budget from Eq. (1) in observations

In this subsection, we describe the methodology used to evaluate the SSL variance equation stemming from Eq. (1) in observations and to obtain Fig. 1. We use the sea level anomaly (SLA) observed by satellite altimetry (AVISO^7^) to approximate the seasonal steric sea level. It has been shown^8^ that seasonal regional variations of sea level are mostly due to the steric sea level and we also show, using the ECCO v4r3 state estimate (more details about this state estimate are given in the following subsection), that this approximation holds almost everywhere at low and mid latitudes except in some semi-enclosed seas (see Fig. S7 of the supplementary file). The AVISO product used has a ${1}/{4}^\circ$ horizontal resolution and we select the period 1993–2014 to be consistent with the analysis performed in the remainder of this article. Following a common practice in oceanography e.g.^27–29^, the mean SLA seasonal cycle is derived by first removing the 1993–2014 trend and then by computing the time mean for each individual month resulting in 12 monthly values. The time tendencies of $\eta$ are obtained by differentiating the daily values of $\eta$ at the start and end of each month divided by the number of days. The mean seasonal cycle of $\eta$ time tendencies is then computed from its monthly time series. The time average of the product between $\eta$ and $\partial _t \eta$ gives Fig. 1a . To obtain Fig. 1b, the net heat flux is first computed from the sum of the net short wave, net long wave, latent and sensible heat fluxes, given by ERA5^25^ on a ${1}/{4}^\circ$ grid. The seasonal cycle of *Q* is extracted following the same procedure as for $\eta$. To compute the term $\overline{\eta \frac{\alpha Q}{\rho _0 C_p}}$ shown in Fig. 1, we use the following coefficients: $\rho _0={1028}\,\hbox {kg}\,\hbox {m}^{-3}$ and $C_p={4000}\,\hbox {J}\,\hbox {kg}^{-1}\,\hbox {K}^{-1}$. The thermal expansion coefficient $\alpha$ is computed from the ECCOv4r3 surface temperature and surface salinity, then time averaged over 1993–2014 as well as zonally averaged so that the resulting coefficient is only a function of latitude. We have also checked (Fig. S8 of the supplementary file) that a different choice of net heat flux database (OAFlux^43^) leads to very similar results.

Note that the methodology used in this article and in many different studies to extract the mean seasonal cycle implies that the product of $\eta$ and ${\partial _t \eta }$ is not exactly zero. The mean seasonal cycle of the time tendencies of $\eta$ is indeed not exactly equal to the time tendencies of the mean seasonal cycle of $\eta$. If the amplitude of the seasonal cycle increases or decreases over the 22 years of the ECCOv4 period, then the product $\overline{\eta \partial _t\eta }$ will reflect the mean rate of this change. Additionally, the various approximations used by AVISO to construct daily SLA on a regular 2D grid from an ensemble of inter-calibrated altimeter missions and the use of SLA to approximate SSL seasonal variations also contribute to the fact that $\eta \frac{\partial \eta }{\partial t}$ is not exactly zero. However, Fig. 1a shows that this term has amplitudes and patterns that are very different from the term linked with the net heat flux (Fig. 1a) and it therefore demonstrates that Eq. (1) is not complete.

### The ECCOv4r3 dataset

Seasonal SSL variance budgets are computed using the ECCOv4r3 state estimate which covers the period 1992–2017. This state estimate is the output of the Massachusetts Institute of Technology general circulation model (MITgcm) assimilating available observations for the period 1992 to 2017^38^. The advantage of ECCOv4 is that it satisfies the equation of motion and conservation laws hence making it possible to compute tracer budgets. The solution used in this article is computed on the LLC90 grid which has an average horizontal resolution of ${1}^\circ$ and 50 vertical levels. Outputs of the model consist of one month average and the closed budget can be obtained for the 1993–2014 period. Thus we compute the SSL budget over this 1993–2014 period. Snapshots at the start and end of each month are also provided by ECCO in order to compute the tracer time tendencies required to close the budgets. ECCO has already been used in several past studies to compute budget of steric sea level variations^27–30^.

For each variable from the monthly model outputs, at each gridpoint, the seasonal cycle is obtained by first removing the linear trend over the 1993–2014 period, and then by computing the 12 monthly values as average of the respective monthly anomalies over the 22 years. The SSL seasonal cycle studied here is thus an average of the seasonal cycle over the 22 years of the 1993–2014 period.

### Seasonal SSL variance budget

The seasonal SSL anomaly $\eta$ is expressed as the vertical integral of the seasonal density anomaly $\rho$ as follows:

6 $$\begin{aligned} \eta =-\frac{1}{\rho _0}\int _{-H}^0 \rho \, \textrm{d}z , \end{aligned}$$

where $\rho _0={1029}\,\hbox {kg}\,\hbox {m}^{-3}$. The evolution equation for $\eta$ is then simply obtained as the time derivative of (6):

7 $$\begin{aligned} \frac{\partial \eta }{\partial t}=-\frac{1}{\rho _0}\int _{-H}^0 \frac{\partial \rho }{\partial t} \textrm{d}z . \end{aligned}$$

Following^30^, the time evolution of $\rho$ can be decomposed into the time evolution of the potential temperature $\theta$ and salinity S as:

8 $$\begin{aligned} \frac{\partial \rho }{\partial t} = -\rho _0\alpha \frac{\partial \theta }{\partial t} + \rho _0 \beta \frac{\partial S}{\partial t} , \end{aligned}$$

where $\alpha =-\frac{1}{\rho _0}\frac{\partial \rho }{\partial \theta }$ is the thermal expansion coefficient and $\beta =\frac{1}{\rho _0}\frac{\partial \rho }{\partial S}$ is the haline contraction coefficient and vary in space and time according to the temperature, salinity and pressure fields. Then, the ECCO V4r3 state estimate gives all the necessary terms to decompose the potential temperature and salinity evolution equation into advection, diffusion and surface fluxes terms (net heat flux for the potential temperature and freshwater flux for the salinity):

9 $$\begin{aligned} \left( \frac{\partial \theta }{\partial t},\frac{\partial S}{\partial t}\right) =\left( {\textrm{adv}}_{\theta },{\textrm{adv}}_{S}\right) +\left( {\textrm{dif}}_{\theta },{\textrm{dif}}_{S}\right) +\left( {\textrm{f}}_{\theta },{\textrm{f}}_{S}\right) \end{aligned}$$

where ${\textrm{adv}}_{\theta },{\textrm{adv}}_{S}$ are respectively the advective terms for temperature and salinity, ${\textrm{dif}}_{\theta },{\textrm{dif}}_{S}$, the diffusive terms for temperature and salinity and ${\textrm{f}}_{\theta },{\textrm{f}}_{S}$, the atmospheric forcing terms for temperature and salinity.

Using this decomposition (i.e. Eq. (9)) of the temperature and salinity evolution equation and Eq. (8), the time evolution of $\rho$ is decomposed itself into advection ${\textrm{adv}}_{\rho }=-\rho _0 \alpha {\textrm{adv}}_{\theta }+\rho _0 \beta {\textrm{adv}}_{S}$, parametrized diffusion ${\textrm{dif}}_{\rho }=-\rho _0 \alpha {\textrm{dif}}_{\theta }+\rho _0 \beta {\textrm{dif}}_{S}$, and buoyancy fluxes from the atmosphere ${\textrm{flu}}_{\rho }=-\rho _0 \alpha {\textrm{f}}_{\theta }+\rho _0 \beta {\textrm{f}}_{S}$:

10 $$\begin{aligned} \frac{\partial \rho }{\partial t}= {\textrm{adv}}_{\rho }+ {\textrm{dif}}_{\rho }+ {\textrm{flu}}_{\rho }. \end{aligned}$$

Then, inserting Eq. (10) in Eq. (7) gives:

11 $$\begin{aligned} \frac{\partial \eta }{\partial t}= {\textrm{adv}}+ {\textrm{dif}}+ {\textrm{flu}}. \end{aligned}$$

where $X={\textrm{adv}},{\textrm{dif}}$, or ${\textrm{flu}}$ is related to $X_{\rho }$ through the following formula:

12 $$\begin{aligned} {X}= -\frac{1}{\rho _0} \int _{-H}^0 X_{\rho } \textrm{d}z. \end{aligned}$$

Following previous work on density variance^31,33,34,44^, temperature variance^45,46^ and also steric sea level variance^30^, multiplying equation (11) by $\eta$ and computing the time average (over all months of the mean seasonal cycle) gives the budget of the seasonal cycle steric sea level variance:

13 $$\begin{aligned} \overline{\eta \frac{\partial \eta }{\partial t}}=\overline{ \frac{1}{2}\frac{\partial \eta ^2}{\partial t}}= & {} \overline{\eta \textrm{adv}} +\overline{\eta \textrm{dif}} +\overline{\eta \textrm{flu}},\nonumber \\= & {} \mathrm {VAR_{adv}} +\mathrm {VAR_{dif}} +\mathrm {VAR_{flu}}, \end{aligned}$$

where the overline represents the average over the seasonal cycle and $\overline{\eta X}$ is a 2D field, it is positive (negative) when $\overline{\eta X}$ is a source (sink) of seasonal SSL variance. By examining the sign and relative intensity of the seasonal SSL variance budget terms, it is then possible to determine which term is locally driving or damping the seasonal variation of the SSL. $\overline{\eta X}$ can be a source (sink) in two cases: 1) if $\eta >0$ and $-\int _{-H}^0 X_{\rho } \textrm{d}z>0$ ($-\int _{-H}^0 X_{\rho } \textrm{d}z<0$) because the last term acts to increase (decrease) the positive anomaly of $\eta$ or 2) if $\eta <0$ and $-\int _{-H}^0 X_{\rho } \textrm{d}z<0$ ($-\int _{-H}^0 X_{\rho } \textrm{d}z>0$) because the last term acts to increase (decrease) the magnitude of the negative anomaly of $\eta$. Note that, similar to the analysis performed on SLA from AVISO, in practice the term $\overline{\frac{\partial \eta ^2}{\partial t}}$ is not exactly zero because the seasonal cycle of the SSL time tendencies is not exactly equal to the time tendencies of the SSL seasonal cycle. However, Figure 3a shows that this term remains one order of magnitude smaller than the other terms.

### Variance budget for the spatially-averaged seasonal steric sea level

In this subsection, we compute the variance budget for the spatially-averaged seasonal steric sea level. To this end, the seasonal steric sea level anomaly $\eta$ is first spatially averaged $\langle \eta \rangle$, where $\langle .\rangle$ represents the spatial average over the Northern hemisphere for latitudes North of a given limit value or over the Southern hemisphere for latitudes South of a given boundary. Then the equation for the evolution of $\eta$ is also spatially averaged over the same region as follows:

14 $$\begin{aligned} \frac{\partial \langle \eta \rangle }{\partial t} = \langle \textrm{adv}\rangle +\langle \textrm{dif}\rangle +\langle \textrm{flu}\rangle \end{aligned}$$

Multiplying this equation by $\langle \eta \rangle$ gives the budget for the variance of $\langle \eta \rangle$:

15 $$\begin{aligned} \overline{ \frac{1}{2}\frac{\partial \langle \eta \rangle ^2}{\partial t}}= & {} \overline{\langle \eta \rangle \langle \textrm{adv}\rangle } +\overline{\langle \eta \rangle \langle \textrm{dif}\rangle } +\overline{\langle \eta \rangle \langle \textrm{flu}\rangle },\nonumber \\= & {} \mathrm {VAR^H_{adv}} +\mathrm {VAR^H_{dif}} +\mathrm {VAR^H_{flu}}, \end{aligned}$$

$\overline{ \frac{1}{2}\frac{\partial \langle \eta \rangle ^2}{\partial t}}$ is the variance tendency term, $\mathrm {VAR^H_{adv}}=\overline{\langle \eta \rangle \langle \textrm{adv}\rangle }$, $\mathrm {VAR^H_{dif}}=\overline{\langle \eta \rangle \langle \textrm{dif}\rangle }$ and $\mathrm {VAR^H_{flu}}=\overline{\langle \eta \rangle \langle \textrm{flu}\rangle }$ are respectively the terms associated with advection, diffusion and surface buoyancy fluxes for the variance equation of the SSL hemispheric average.

### Vertically-averaged density and $\mathrm {VAR_{flu}}$

Inserting Eqs. (6) in (12), and recognizing that the atmospheric density flux is mostly due to the net atmospheric heat flux *q* (in $\hbox {W}\,\hbox {m}^{-3}$) (see supporting information Fig. S2), $\mathrm {VAR_{flu}}$ can be written as:

16 $$\begin{aligned} \mathrm {VAR_{flu}}=\overline{\eta \textrm{flu}}= \frac{1}{\rho _0^2} \overline{\int _{-H}^0\rho \, \textrm{d}z \int _{-H}^0 -\frac{\alpha }{C_p} q \textrm{d}z} . \end{aligned}$$

Then, defining the vertically-averaged density anomaly as $\rho _{\textrm{BT}}=\frac{1}{H}\int _{-H}^0\rho \textrm{d}z$ and the vertically-averaged net heat flux as $Q_{\textrm{BT}}=\frac{1}{H}\int _{-H}^0 -\frac{\alpha }{C_p} q \textrm{d}z$ (*q* is non-zero below the surface because of the penetrating nature of the shortwave term) in this equation one recovers Eq. (4).

### Advective term decomposition

To decompose the advective term in the seasonal SSL budget, we first decompose the advective term in the density evolution equation (10) into the contributions of laminar (resolved) velocities and eddy-induced velocities linked to the Gent and McWilliams parameterization:

17 $$\begin{aligned} {\rm adv}_{\rho }={\rm adv}_{\rho }^{\rm laminar}+{\rm adv}_{\rho }^{\rm eddy}, \end{aligned}$$

where $\textrm{adv}_{\rho}^{\textrm{laminar}}$ is the ECCO v4r3 resolved part of the seasonal advective term and $\textrm{adv}^{\textrm{eddy}}_{\rho}$ the eddy induced part ot the seasonal advective term. Using Eq. (17), $\mathrm {VAR_{adv}}$=$\overline{\eta \textrm{adv}}$ can thus be decomposed into two terms $\mathrm {VAR^{laminar}_{adv}}$=$\overline{\eta \textrm{adv}_{\textrm{laminar}}}$ and $\mathrm {VAR^{eddy}_{adv}}$=$\overline{\eta \textrm{adv}_{\textrm{eddy}}}$, as shown by formula (5) where the notation $\mathrm {VAR^{X}_{adv}}$=$\overline{\eta \textrm{adv}_{X}}$ is given by:

18 $$\begin{aligned} \mathrm {VAR^{X}_{adv}}=\overline{\eta \textrm{adv}_{X}}= \overline{-\frac{\eta }{\rho _0} \int _{-H}^0 \textrm{adv}^X_{\rho } \textrm{d}z} , \end{aligned}$$

where *X* stands for $\textrm{laminar}$ or $\textrm{eddy}$.

### Supplementary Information

Supplementary Information.
