# Supplementary material for: Advection surface-flux balance controls the seasonal steric sea level amplitude
Source: Sci Rep. 2024 May 9;14:10644. doi: 10.1038/s41598-024-61447-y (PMC11082253; doi:10.1038/s41598-024-61447-y)
Supplement: Supplementary file 1 — Supplementary Information. [file 41598_2024_61447_MOESM1_ESM.pdf]

Supplementary file for: "Advection-Surface Flux Balance controls the Seasonal Steric Sea Level Amplitude"

Antoine Hochet[1*], William Llovel[1], Thierry Huck[1] and Florian Sévellec[1]

[1*]Univ Brest, CNRS, IFREMER, IRD, Laboratoire d'Océanographie Physique et Spatiale (LOPS), IUEM, Brest, France.

*Corresponding author(s). E-mail(s): antoine.hochet@univ-brest.fr;

1 Supplementary Figures

1.1 Figure S1

In Figure S1 of this supplementary file, we show the time mean of the product between the seasonal cycle of Sea Surface Temperature (SST) and the seasonal cycle of the net heat flux i.e. $\overline{\mathrm{SST}\frac{Q}{\rho_0 C_p}}$ where ρ_0 is a reference density with $\rho_0 = 1029\,\mathrm{kg\,m^{-3}}$ and $C_p = 4000\,\mathrm{J\,kg^{-1}\,K^{-1}}$ the specific heat of seawater. The seasonal cycle of the SST, used in Figure (S1) is obtained from the NOAA OI SST v2 dataset[1], which is available on a 1/4° global grid from 1981. We first select the period 1993-2014, corresponding to ECCO v4r3, remove the trends, the time mean, and compute the seasonal cycle from the monthly time series. The net heat flux is computed from the sum of the net short wave, net long wave, latent and sensible heat fluxes, given by ERA5 [2] on a 1/4° grid. Figure S1 shows that $\overline{\mathrm{SST}\frac{Q}{\rho_0 C_p}}$ is positive everywhere, demonstrating that on seasonal time scales the net heat flux acts to increase the amplitude of the seasonal cycle of SST. This is in strong contrast to the results obtained for the seasonal cycle of steric sea level (Figure 1 of the main manuscript), which is negative over about half of the ocean surface.

1.2 Figure S2

In Figure S2 of this supplementary file, the buoyancy flux term of the SSL seasonal cycle variance budget ($\mathrm{VAR_{flu}}$) is decomposed into a part linked with the net heat flux (${\mathrm{VAR_{flu}}}^{\theta}$) and a part linked with freshwater fluxes (${\mathrm{VAR_{flu}}}^{S}$), that is:

$$\mathrm{VAR_{flu}} = {\mathrm{VAR_{flu}}}^{\theta} + {\mathrm{VAR_{flu}}}^{S} \tag{1}$$

$\mathrm{VAR_{flu}}$ is almost everywhere controlled by the net heat flux variations (Fig. S2**a**). The freshwater fluxes term ${\mathrm{VAR_{flu}}}^{S}$ (Fig. S2**b**) has its largest magnitude at low latitudes in both hemispheres and can either be positive (a source of SSL seasonal cycle variance) or negative (a sink). When globally averaged, both terms are positive: $0.1\,\mathrm{cm^2\,yr^{-1}}$ for ${\mathrm{VAR_{flu}}}^{S}$ and $0.8\,\mathrm{cm^2\,yr^{-1}}$ for ${\mathrm{VAR_{flu}}}^{\theta}$, and confirm that ${\mathrm{VAR_{flu}}}^{\theta}$ is the largest contributor to $\mathrm{VAR_{flu}}$ which has a global average of $0.9\,\mathrm{cm^2\,yr^{-1}}$.

1.3 Figure S3

In Figure S3 we show the comparison between the seasonal cycle of the SSL time tendencies ($\frac{\partial\eta}{\partial t}$) and the seasonal cycle of the term linked with the net heat flux ($\frac{\alpha Q}{\rho_0 c_p}$). Results are averaged North of 30°N in order to select only the Northern hemisphere phase and remove low latitudes where the phase is less clear (see Fig. 2a,b). Note that we have checked that results presented below also hold for the Southern hemisphere. The average is computed for regions where $\frac{\alpha Q}{\rho_0 c_p} > 0$ (Fig. S3a), $\frac{\alpha Q}{\rho_0 c_p} < 0$ (Fig. S3 b) and for regions where $\frac{\alpha Q}{\rho_0 c_p} > 10\,\mathrm{cm^2\,yr^{-1}}$ (Fig. S3c), $\mathrm{VAR_{flu}} < -10\,\mathrm{cm^2\,yr^{-1}}$ (Fig. S3d). In this figure we see that $\frac{\partial\eta}{\partial t}$ occurs before $\frac{\alpha Q}{\rho_0 c_p}$ in regions where $\mathrm{VAR_{flu}} > 0$ (Fig. S3a) while it occurs after $\frac{\alpha Q}{\rho_0 c_p}$ in regions where $\mathrm{VAR_{flu}} < 0$ (Fig. S3b). This time lag of approximately 2 weeks becomes larger (up to 3 weeks) when the average is computed over regions where $|\mathrm{VAR_{flu}}| > 10\,\mathrm{cm^2\,yr^{-1}}$. (Fig. S3c,d). This relatively small time lag is sufficient to induce a large SSL variance flux with magnitudes $\sim 30\,\mathrm{cm^2\,yr^{-1}}$ (see Fig. 1 of the main manuscript).

1.4 Figure S4

In Figure S4 of this supplementary file, we compute the steric sea level variance budget using all frequencies of the ECCO state estimate. The budget equation is similar to equation (2) of the main manuscript (see also section Method):

$$\frac{1}{2}\frac{\partial\left(\eta^{\mathrm{all}}\right)^2}{\partial t} = \mathrm{VAR^{all}_{adv}} + \mathrm{VAR^{all}_{dif}} + \mathrm{VAR^{all}_{flu}} \tag{2}$$

The only difference with Eq. (2) from the main manuscript is that instead of using the mean seasonal cycle of every term, we now use the detrended anomalies of each term. The superscript ^{all} in Eq. (2) indicates that each term

contain all frequencies including interannual, annual and subannual. Fig. S4 shows each of the four terms of this variance budget. The results are very similar to the results obtained for the mean seasonal cycle (Fig. 3 a,b,c,d). The main balance is indeed between $\mathrm{VAR}_{\mathrm{flu}}^{\mathrm{all}}$ and $\mathrm{VAR}_{\mathrm{adv}}^{\mathrm{all}}$ and sources and sinks regions follows the same pattern as in Fig. 3. The main differences are located close to the equator in the Pacific ocean. The global average of each term is however larger when all frequencies are included. The fact that the variance budget terms are similar when all frequencies are included and when only the mean seasonal cycle is considered means that the SSL variance fluxes are dominated by the seasonal cycle. Moreover it shows that the methodology we use to extract the mean seasonal cycle (time mean of the monthly time series) is able to successfully extract the main characteristics of the seasonal cycle.

1.5 Figure S5

In Fig. S5, we show the southern and northern hemisphere averages of the budget for the seasonal variations of the SSL given by Eq. (14) of the main manuscript. In both hemispheres, the term associated with the surface buoyancy flux (mainly due to the net heat flux) is close to the seasonal trends of the SSL, but present a small time lag, of the order of a few weeks which implies a positive SSL variance flux of $5\,\mathrm{cm}^2\,\mathrm{yr}^{-1}$ in the NH and $3\,\mathrm{cm}^2\,\mathrm{yr}^{-1}$ in the SH. The advective term $<\mathrm{adv}>$ has a weaker amplitude than the $<\mathrm{flu}>$ term ($\sim$5 cm yr^{-1} vs. $\sim$15 cm yr^{-1} in the NH), but is almost opposite to the hemispheric variations of $<\eta>$ and acts to balance the effect of the surface buoyancy flux in both hemispheres.

1.6 Figure S6

In Fig. S6, we compute the term linked with the buoyancy fluxes in the SSL variance budget ($\mathrm{VAR}_{\mathrm{flu}}$) assuming that there is no density anomalies below the surface layer, that is:

$$\mathrm{VAR}_{\mathrm{flu}}{}^{\mathrm{SURF}} = \overline{\frac{1}{\rho_0^2}\int_{-h}^{0} \rho\,\mathrm{d}z \int_{-H}^{0} \mathrm{flu}_\rho\,\mathrm{d}z} \tag{3}$$

where $h = 10\,\mathrm{m}$ and corresponds to the first layer of the ECCO V4r3 state estimate, and where flu_ρ is the buoyancy forcing term of the density evolution equation (see section "Seasonal SSL variance budget" in Methods). $\mathrm{VAR}_{\mathrm{flu}}{}^{\mathrm{SURF}}$ is almost everywhere positive (Fig. S6), showing that the seasonal cycle density anomalies at the surface are in phase with the surface buoyancy flux seasonal cycle.

1.7 Figure S7

In Figure S7, we assess the differences between the seasonal variations of the steric sea level (η) and the seasonal variations of sea surface anomalies (SSA). SSA is corrected from the global mean sea level so that the difference between η and SSA is the manometric sea level, i.e.:

$$SSA - \eta = \underbrace{\frac{P_b}{\rho_0 g}}_{\text{Manometric sea level}} \tag{4}$$

SSA and η are obtained from the ECCO v4r3 state estimate for the period 1993-2014. The seasonal cycle is obtained by first removing the trend and then by computing the time average for each individual month resulting in a 12 points time series. Figure S7 shows the standard deviation of η (left panel) and the standard deviation of the difference between SSA and η. At low and mid latitudes the manometric component of the seasonal sea level is generally negligible compared to the steric sea level except in several semi-enclosed regions such as the Arabian sea or the Yellow sea. At high latitudes, particularly in the Arctic ocean, the standard deviation of the manometric sea level becomes important and cannot be neglected.

1.8 Figure S8

In Figure S8, we reproduce Figure 1b of the main manuscript i.e. $\overline{\eta \frac{\alpha Q}{\rho_0 C_p}}$ but using the net heat flux Q_{OAflux} from the Woods Hole Oceanographic Institute Objectively Analyzed air-sea Fluxes for the global oceans (OAFlux) [3] instead of ERA-5 from ECMWF. OAFlux uses objective analysis to obtain optimal estimates of flux-related surface meteorology and then computes the global fluxes by using the state-of-the-art bulk flux parameterizations [4]. The OAFlux net heat flux used here has a 1° horizontal resolution, is available over the period 1993-2009 and is interpolated on the AVISO 1/4° grid. As before, the mean seasonal cycle is extracted from Q_{OAflux} by first removing the trend and then by computing the time mean over each individual month. Figure S8 shows that using the OAflux net heat flux seasonal cycle extracted to compute $\overline{\eta \frac{\alpha Q}{\rho_0 C_p}}$ gives similar results to the one obtained from ERA-5. Differences (Fig. S8b) are almost everywhere one order of magnitude smaller than the original signal, largest values are found in eddy rich regions but this is likely due to the coarse 1° resolution of OAFlux compared to the 1/4° of ERA-5. We conclude that our estimate of $\overline{\eta \frac{\alpha Q}{\rho_0 C_p}}$ is robust.

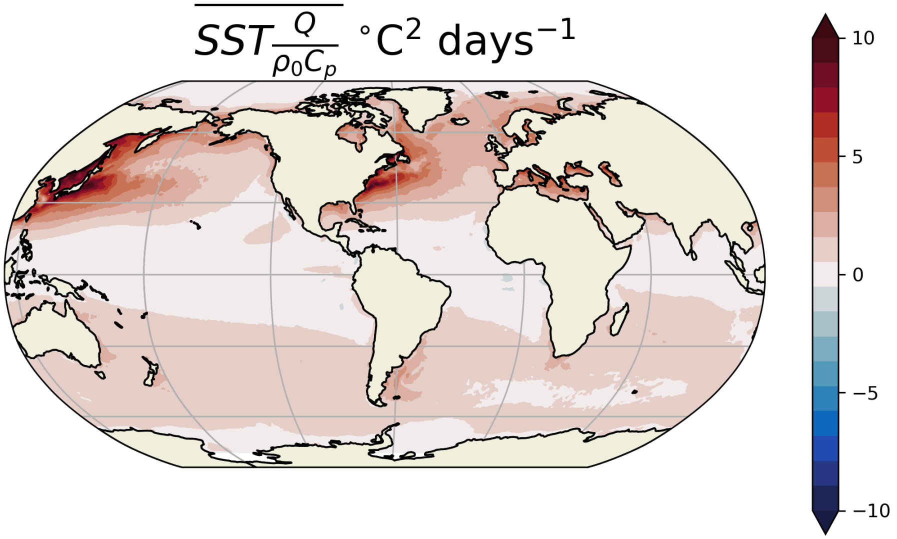

Fig. S1 The time mean of the product between the seasonal variations of net heat flux and SST is everywhere positive. SST is obtained from the NOAA OI SST v2 dataset and net heat flux from the ERA5 reanalysis. Units are in $^\circ$C^2 days^{-1}.

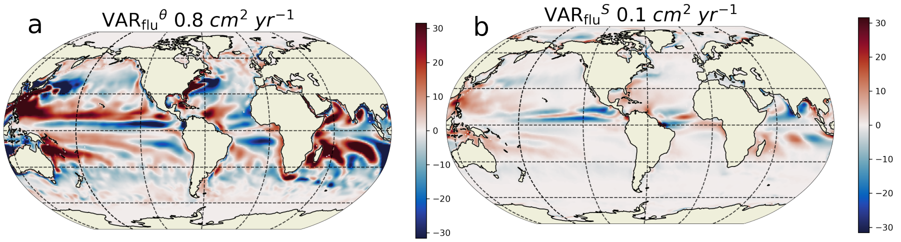

Fig. S2 The buoyancy forcing $\mathrm{VAR_{flu}}$ **term of the SSL seasonal cycle variance budget is controlled by the net heat flux.** (a) net heat flux contribution (in $\mathrm{cm^2\,yr^{-1}}$) $\mathrm{VAR_{flu}}^{\theta}$ and (b) freshwater flux contribution $\mathrm{VAR_{flu}}^{S}$. The global average of each term is given in each title.

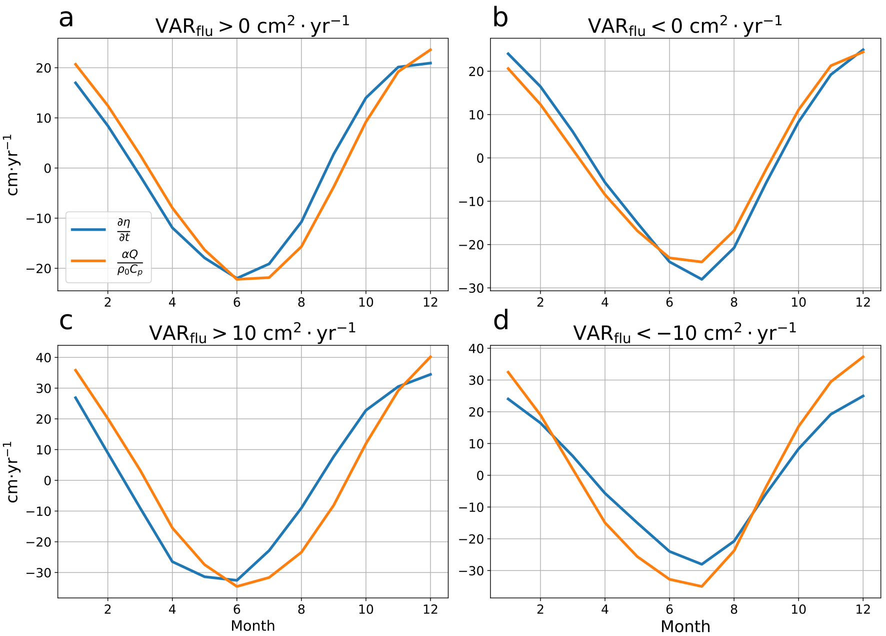

Fig. S3 A non negligible time lag is found between the seasonal cycle of the SSL time tendencies ($\frac{\partial \eta}{\partial t}$) and the seasonal cycle of the net heat flux term ($\frac{\alpha Q}{\rho_0 c_p}$). The seasonal cycle of the net heat flux (orange lines) and time tendencies of SSL (blue lines) are averaged North of 30°N in regions where $\mathrm{VAR_{flu}} > 0$ (panel a) and $\mathrm{VAR_{flu}} < 0$ (panel b) and in regions where $\mathrm{VAR_{flu}} > 10\,\mathrm{cm^2\,yr^{-1}}$ (panel c) and $\mathrm{VAR_{flu}} < -10\,\mathrm{cm^2\,yr^{-1}}$ (panel d).

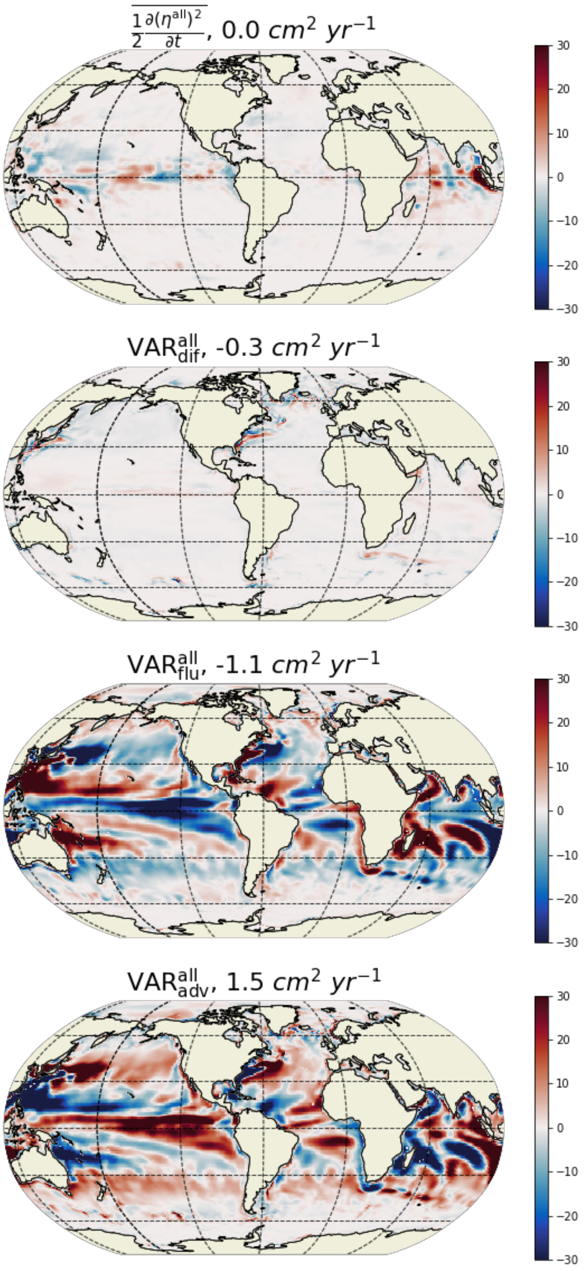

Fig. S4 The SSL variance budget is similar to the SSL seasonal cycle variance budget. Same as Figure 3 a,b,c,d from the main manuscript except that the variance is computed from the monthly outputs, such that all frequencies are used (interannual, annual and subannual) to compute the budget and the seasonal cycle is not extracted.

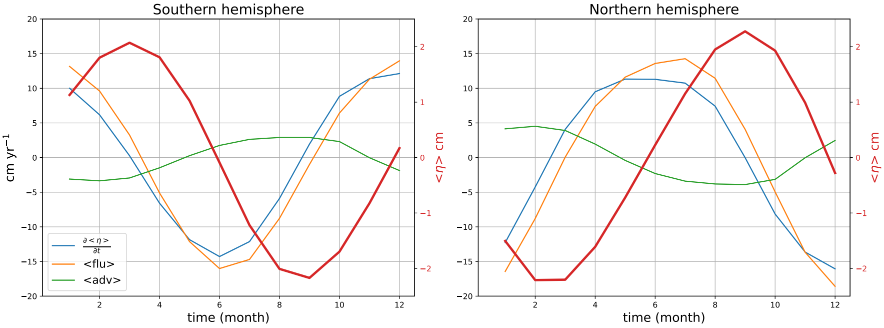

Fig. S5 Hemispheric horizontal average of the seasonal SSL budget terms. Left panel: southern hemisphere, right panel: northern hemisphere. The red line represents the hemispheric variations of the SSL (in cm), the blue, orange and green lines respectively show the hemispheric horizontal average of the time tendencies of the SSL, buoyancy surface flux and advection. Diffusion is not shown because it is negligible. Budget terms are in cm yr^{-1}.

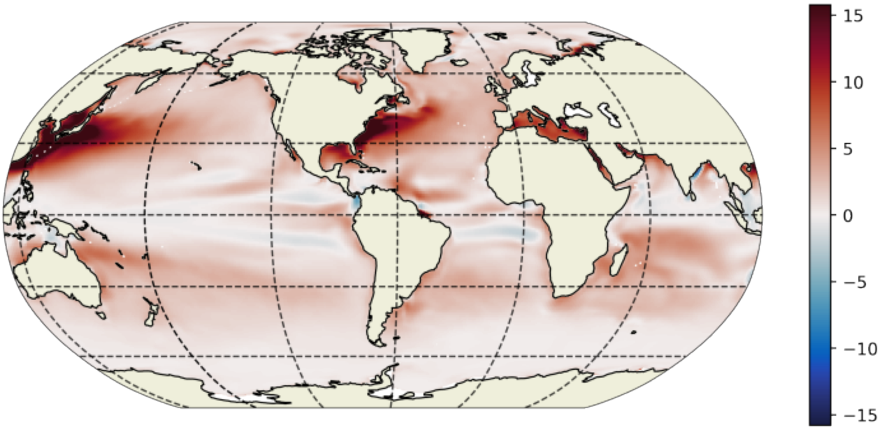

Fig. S6 The seasonal cycle of buoyancy forcing is in phase with surface density anomalies almost everywhere. Term $\mathrm{VAR_{flu}}^{\mathrm{SURF}}$ (in $\mathrm{cm^2\,yr^{-1}}$) which is the same as $\mathrm{VAR_{flu}}$ except that sub-surface density anomalies are set to zero (see text 1.6).

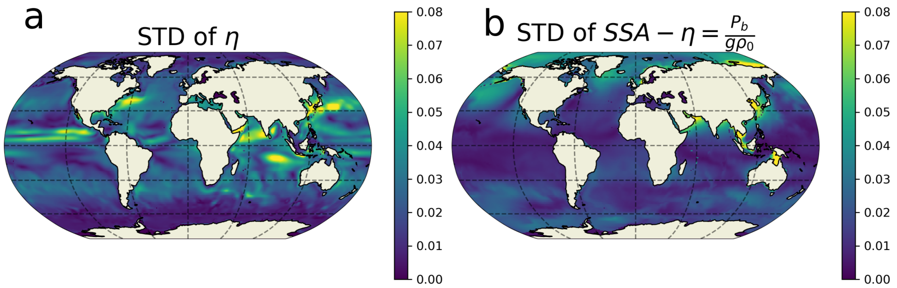

Fig. S7 Seasonal steric sea level is well approximated by total sea level except at high latitudes and in several semi-enclosed seas. (a) standard deviation of the seasonal steric sea level (η in m) obtained in ECCO, (b) standard deviation of the difference between the total seasonal sea level anomalies ($SSA - \eta$ in m) and the steric sea level.

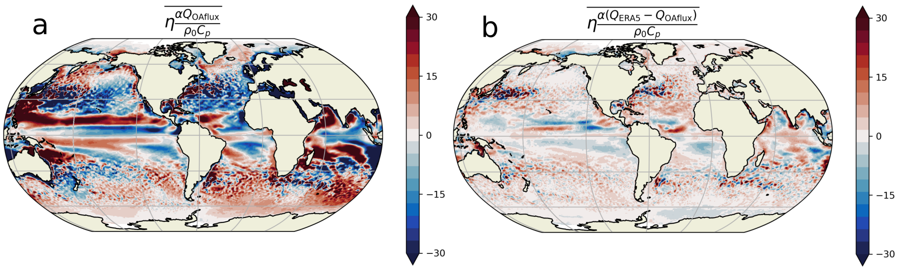

Fig. S8 The estimate of $\overline{\eta \frac{\alpha Q}{\rho_0 C_p}}$ is robust to the choice of net heat flux dataset. Estimate (in $\mathrm{cm}^2\,\mathrm{yr}^{-1}$) using the net heat flux values derived from the OAFlux dataset (a) and differences between the ERA-5 and OAFlux estimates (b).

References

[1] Huang, B., Liu, C., Banzon, V., Freeman, E., Graham, G., Hankins, B., Smith, T., Zhang, H.-M.: Improvements of the daily optimum interpolation sea surface temperature (doisst) version 2.1. Journal of Climate **34**(8), 2923–2939 (2021)

[2] Hersbach, H., Bell, B., Berrisford, P., Hirahara, S., Horányi, A., Muñoz-Sabater, J., Nicolas, J., Peubey, C., Radu, R., Schepers, D., *et al.*: The ERA5 global reanalysis. Quarterly Journal of the Royal Meteorological Society **146**(730), 1999–2049 (2020)

[3] Yu, L., Weller, R.A.: Objectively analyzed air–sea heat fluxes for the global ice-free oceans (1981–2005). Bulletin of the American Meteorological Society **88**(4), 527–540 (2007)

[4] OAflux. https://oaflux.whoi.edu/. Accessed: 2023-07-25
